# Evaluation of the Radiomics Method for the Prediction of Atypical Adenomatous Hyperplasia in Patients With Subcentimeter Pulmonary Ground-Glass Nodules

**DOI:** 10.3389/fonc.2021.698053

**Published:** 2021-08-05

**Authors:** Bin Wang, Preeti Hamal, Xue Meng, Ke Sun, Yang Yang, Yangyang Sun, Xiwen Sun

**Affiliations:** ^1^Department of Radiology, Shanghai Pulmonary Hospital, Tongji University School of Medicine, Shanghai, China; ^2^Department of Radiology, Shanghai East Hospital, Tongji University School of Medicine, Shanghai, China

**Keywords:** lung neoplasms, radiomics, tomography; spiral computed, forecasting, thoracic surgery

## Abstract

**Objectives:**

We aimed to develop a prediction model to distinguish atypical adenomatous hyperplasia (AAH) from early lung adenocarcinomas in patients with subcentimeter pulmonary ground-glass nodules (GGNs), which may help avoid aggressive surgical resection for patients with AAH.

**Methods:**

Surgically confirmed cases of AAH and lung adenocarcinomas manifesting as GGNs of less than 1 cm were retrospectively collected. A prediction model based on radiomics and clinical features identified from a training set of cases was built to differentiate AAH from lung adenocarcinomas and tested on a validation set.

**Results:**

Four hundred and eighty-five eligible cases were included and randomly assigned to the training (*n* = 339) or the validation sets (*n* = 146). The developed radiomics prediction model showed good discrimination performance to distinguish AAH from adenocarcinomas in both the training and the validation sets, with, respectively, 84.1% and 82.2% of accuracy, and AUCs of 0.899 (95% CI: 0.867–0.931) and 0.881 (95% CI: 0.827–0.936).

**Conclusion:**

The prediction model based on radiomics and clinical features can help differentiate AAH from adenocarcinomas manifesting as subcentimeter GGNs and may prevent aggressive resection for AAH patients, while reserving this treatment for adenocarcinomas.

## Introduction

The detection rate of lung ground-grass nodules (GGNs) is increasing rapidly, many of which are identified as atypical adenomatous hyperplasia (AAH), whereas others are early lung adenocarcinomas (1, 2). Determining appropriate timing of surgical intervention for treatment of neoplastic GGNs represents a big challenge in clinics. Lung AAH consists of proliferating type II alveolar pneumocytes and/or Clara cells, which display different cellular and molecular manifestations compared to lung adenocarcinomas (3, 4), and is usually considered as a precancerous lesion (5, 6). Yet, in clinical practice, AAH nodules are also considered benign. Therefore, the patients with AAH may need different treatment strategies than those with adenocarcinomas and require a follow-up approach rather than surgery. Nevertheless, a large part of the patients with AAH have undergo surgical treatment because of the difficulty to discriminate between AAH and adenocarcinomas based solely on preoperative CT images. Thus, accurate diagnosis of AAH *versus* neoplastic GGNs is essential to decision-making on the treatment to provide to the patients and would help surgeons determine which patients with GGNs should receive surgical treatment and avoid unnecessary or premature surgeries.

Radiomics is a new method that can transform medical images into large numbers of detailed quantitative tumors features, at histologic, cytologic, molecular, and even genetic levels (7–10). The use of radiomics allows doctors to get not only conventional measurements and eye observations, but also abundant microscale quantitative features of the tumor lesions that can help provide precision diagnosis for the patients (11–14). In addition, much research already used radiomics to assist the pathological classification of lung adenocarcinomas. Many of these studies demonstrated that the radiomics method had a better diagnostic performance than radiologists using traditional medical imaging methods (15–17).

Although many studies developed tools for the prediction of lung adenocarcinomas based on radiomics, there has been no research aiming at distinguishing AAH from lung adenocarcinomas. However, accurate discrimination between AAH and early lung adenocarcinomas is of utmost significance to help doctors determine whether a patient is suitable for follow-up or surgical treatment. In this study, we attempted to differentiate AAH from adenocarcinomas manifesting as subcentimeter GGNs. To our knowledge, this is the first study aiming to predict AAH using radiomics method, and we hope that the introduction of our precise predictive approach will help personalized management for patients with malignant GGNs.

## Materials and Methods

### Patients

This study was approved by the institutional review board of our hospital. Owing to the retrospective nature of this research, the provision of informed consent form signed by the patients was waived.

The hospital electronic medical records and radiology working system were searched for cases of lung GGNs, identified as AAH and early lung adenocarcinomas by surgeries between January 2018 and October 2019, and complying with the inclusion criteria. Since AAH is generally considered benign and follow-up treatment is usually recommended, the AAH cases identified by surgeries were a lot fewer than the cases of early-stage lung adenocarcinomas, including adenocarcinoma *in situ*, minimally invasive adenocarcinoma, and invasive adenocarcinoma. To get similar numbers of AAH and adenocarcinomas cases, all AAH cases and 30% of the adenocarcinoma cases, selected by stratified sampling according to the pathological types, were included in the preliminary data of our study. These preliminary data were further selected against the exclusion criteria. The inclusion and exclusion criteria are as follows.

The inclusion criteria were (i) preoperative CT examination performed within 1 month before the surgery; (ii) GGNs’ maximum diameter ≤1 cm; (iii) CT image layer thickness <2 mm; (iv) peripheral GGNs; and (v) confirmed as adenocarcinomas or AAH by surgery. The exclusion criteria were (i) obvious artifacts around the nodules on CT images; (ii) injected with contrast medium for CT; and (iii) tightly connected with the pleura.

The demographics and clinical data of the patients (e.g., age and gender) were also collected. Finally, the included nodules were assigned to a training or a validation set at a 7:3 ratio. The study flowchart is shown in **Supplementary Figure S1**.

### CT Image Acquisition

The preoperative CT examinations were conducted at deep inspiration to avoid the influence of respiratory artifacts. The scanned images were acquired on a Brilliance 40 scanner (Philips Medical Systems, Netherlands) and a Somatom Definition AS scanner (Siemens Medical Systems, Germany). The CT scan parameters and conditions were as follows: 120 kV, 180–220 mAs, 64 × 0.625 mm or 40 × 0.625 mm detector, 0.4 or 1.0 pitch, 512 × 512 matrix, reconstructed at 1.0 mm thickness with 0.7 mm increment, and a standard soft tissue kernel.

### Pathologic Diagnosis

The final pathologic classification was based on histological diagnosis from postoperative paraffin sections. Most diagnoses were made by two pathologists. In case of disagreement, a third senior pathologist was invited to participate to the diagnosis of the disputed case. The results were reported according to the classification of lung adenocarcinomas made by the International Association for the Study of Lung Cancer/American Thoracic Society/European Respiratory Society in 2011.

### Segmentation of Lung Nodules

To extract the region of interest (ROI) from the CT images, a segmentation was performed using the 3D-slicer software (version of 4. 11). Additional manual corrections were conducted by a radiologist with 6 years of experience in the diagnosis of chest imaging and reviewed by another radiologist with 20 years of experience in diagnosis based on chest imaging.

### Extraction of Radiomics Features

The radiomic features were extracted from the segmented ROI area and classified into three categories: intensity, shape, and texture features. Except for the shape features, all features could be obtained from one or several filters, including wavelet, square root, square, gradient, logarithm, gaussian Laplace (LoG) filters, and exponential filters (18).

### Radiomics Features Selection

In the training set, the selection of radiomics features was conducted before the model construction. A Kruskal–Wallis test was used to investigate the relationship between the features and the pathological diagnosis of the selected nodules. A correlation matrix was used to remove redundant features. The absolute columnar mean correlation (CWAAC) was calculated for each feature. When the correlation coefficient of each pair exceeded the 0.8 threshold, the feature was considered with high CWAAC value and was removed.

Finally, intra- and interclass correlation coefficients (ICCs) were used to estimate the intra- and interobserver reproducibility of the radiomics feature extraction. We randomly chose 50 cases for segmentation and feature extraction. The ROI segmentation of these 50 cases was conducted by two radiologists. Then, the first radiologists repeated the same process after 1 week. ICC > 0.75 indicated good agreement of the feature extractions (12).

### Construction of the Radiomics Prediction Model

After selection of the radiomics features, the prediction model was constructed by a random forest method using the training set and tested with the validation set. The ROC curves, AUC value, accuracy, specificity, positive predictive value (PPV), and negative predictive value (NPV) were used to evaluate the predictive performance of the established radiomics model.

### Statistical Analysis

The quantitative clinical and radiomics characteristics are shown as mean ± standard deviation or median [25th–75th]; qualitative characteristics are shown as *n* (%). Comparisons of qualitative features were achieved by the Chi-square test or Fisher exact test, whereas comparisons of quantitative features were performed using a *t*-test or Wilcoxon test. Differences reaching a *P* value < 0.05 by two-tailed tests were considered statistically significant. The statistical analysis was performed with R statistical software (version 3.3.1) and SPSS software (version 20.0).

## Results

### Clinical Information

**Figure 1:**
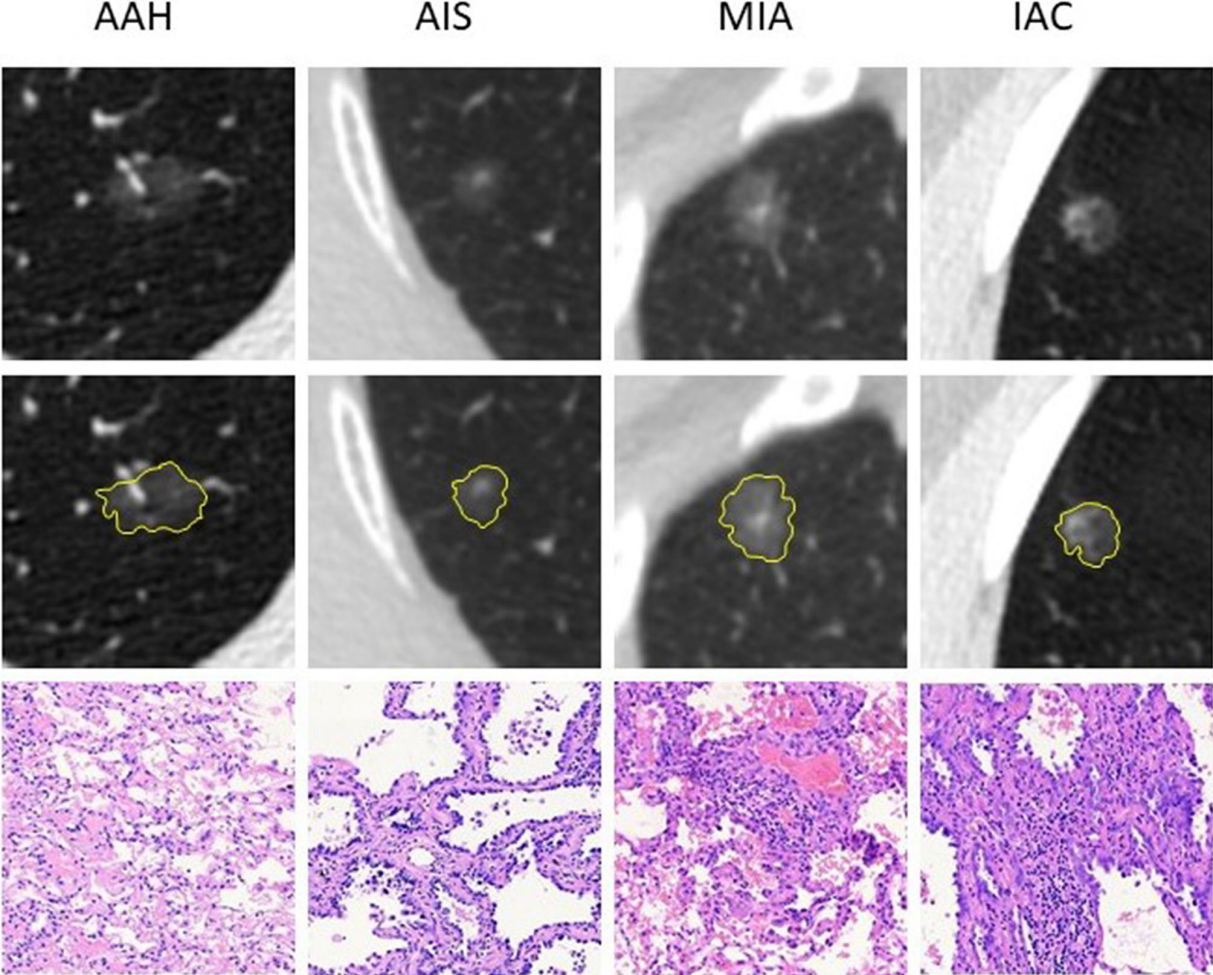
Examples of the included nodules. The yellow shadow represents the shape of the lesions. The pathological pictures are from paraffin sections [hematoxylin and eosin (H&E), ×100 for AAH, AIS, and MIA; H&E, ×40 for IAC]. AAH, atypical adenomatous hyperplasia; AIS, adenocarcinoma *in situ*; MIA, minimally invasive adenocarcinoma; IAC, invasive adenocarcinoma.

The initial search retrieved 1,417 nodules matching the inclusion criteria, of which 190 were AAH cases and 1,227 were adenocarcinoma cases. We randomly selected 30% of the adenocarcinoma cases and all AAH cases for our primary study. Then, according to the exclusion criteria, 17 AAH and 56 adenocarcinomas cases were excluded. Six AAH and 21 adenocarcinomas cases had obvious respiration artifacts around the nodules, 4 AAH and 16 adenocarcinomas cases had been injected with contrast medium for CT, and 7 AAH and 19 adenocarcinomas cases were tightly connected with the pleura. In total, 485 nodules, including 173 AAH cases, 193 AIS cases, 99 MIA cases, and 20 IAC cases from 443 patients were included in our final study. Examples of selected nodules are shown in **Figure 1**. For every 10 modules, 7 were randomly assigned to the training set, and the remaining 3 were assigned to the validation set (7:3 ratio). The process of random selection was performed according to the stratified sampling method based on pathological results. Eventually, 121 AAH and 218 adenocarcinoma cases constituted the training set, and 52 AAH and 94 adenocarcinoma cases constituted the validation set. Detailed information related to the selected nodules is shown in **Table 1**. The study flowchart is shown in **Supplementary Figure S1**.

**Table 1:** Clinical information of the cases in the training and the validation sets.

Clinical features	Training set			Validation set		
	AAH (*n* = 121)	Adenocarcinomas (*n* = 218)	*p*	AAH (*n* = 52)	Adenocarcinomas (*n* = 94)	*p*
Age (years)	50.2 ± 10.1	48.6 ± 11.5	0.193	51.8 ± 11.3	49.5 ± 10.3	0.208
Gender			0.296			0.410
Male	41 (33.9)	62 (28.4)		16 (30.8)	23 (24.5)	
Female	80 (66.1)	156 (71.6)		36 (69.2)	71 (75.5)	
Nodule type			<0.01			<0.05
Pure GGNs	36 (29.8)	33 (15.1)		19 (36.5)	20 (21.3)	
Part solid GGNs	85 (70.2)	185 (84.9)		33 (63.5)	74 (78.7)	
Location (lobe)			0.253			0.246
Upper right	42 (34.7)	71 (32.6)		14 (26.9)	22 (23.4)	
Middle right	13 (10.7)	32 (14.7)		6 (11.5)	13 (13.8)	
Lower right	41 (33.9)	57 (26.1)		18 (34.6)	23 (24.5)	
Upper left	18 (14.9)	49 (22.5)		9 (17.3)	31 (33.0)	
Lower left	7 (5.8)	9 (4.1)		5 (9.6)	3 (5.3)	
Long diameter	9.0 [7.5, 10.2]	9.8 [-8.7, 11.0]	<0.001	8.2 [7.1, 9.2]	9.9 [9.0, 11.2]	<0.001
Short diameter	6.1 [5.5, 7.1]	6.7 [5.9, 7.6]	<0.001	5.9 [4.9, 6.5]	6.7 [5.8, 7.5]	<0.001
Mean CT value	−666.5 [−698.7, −629.8]	−591.8 [−651.5, −540.7]	<0.001	−670.3 [−697.6, −642.1]	−595.7 [−643.1, −533.3]	<0.001
Maximum CT value	−574.0 [−655.0, −494.5]	412.0 [−548.5, −289.8]	<0.001	−580.0 [−649.8, −507.8]	−404 [−504.7, −284.8]	<0.001
Minimum CT value	−800 [−800, −786]	−800 [−800, −768.0]	<0.001	−800 [−800, −789.0]	−800 [−800, −764.3]	<0.001
Variance of CT value	60.8 [39.2, 85.9]	105.5 [71.6, 135.8]	<0.001	59.9 [43.8, 77.5]	109.2 [79.7, 141.7]	<0.001
Volume	219.9 [143.8, 302.0]	281.8 [215.8, 399.5]	<0.001	176.4 [120.7, 246.4]	300.5 [207.6, 404.9]	<0.001

Mean CT value, maximum CT value, minimum CT value, and variance of CT value were measured in the largest circle within the lesion at the maximum cross-section.

AAH, atypical adenomatous hyperplasia.

### Selection of the Radiomics Features

**Figure 2:**
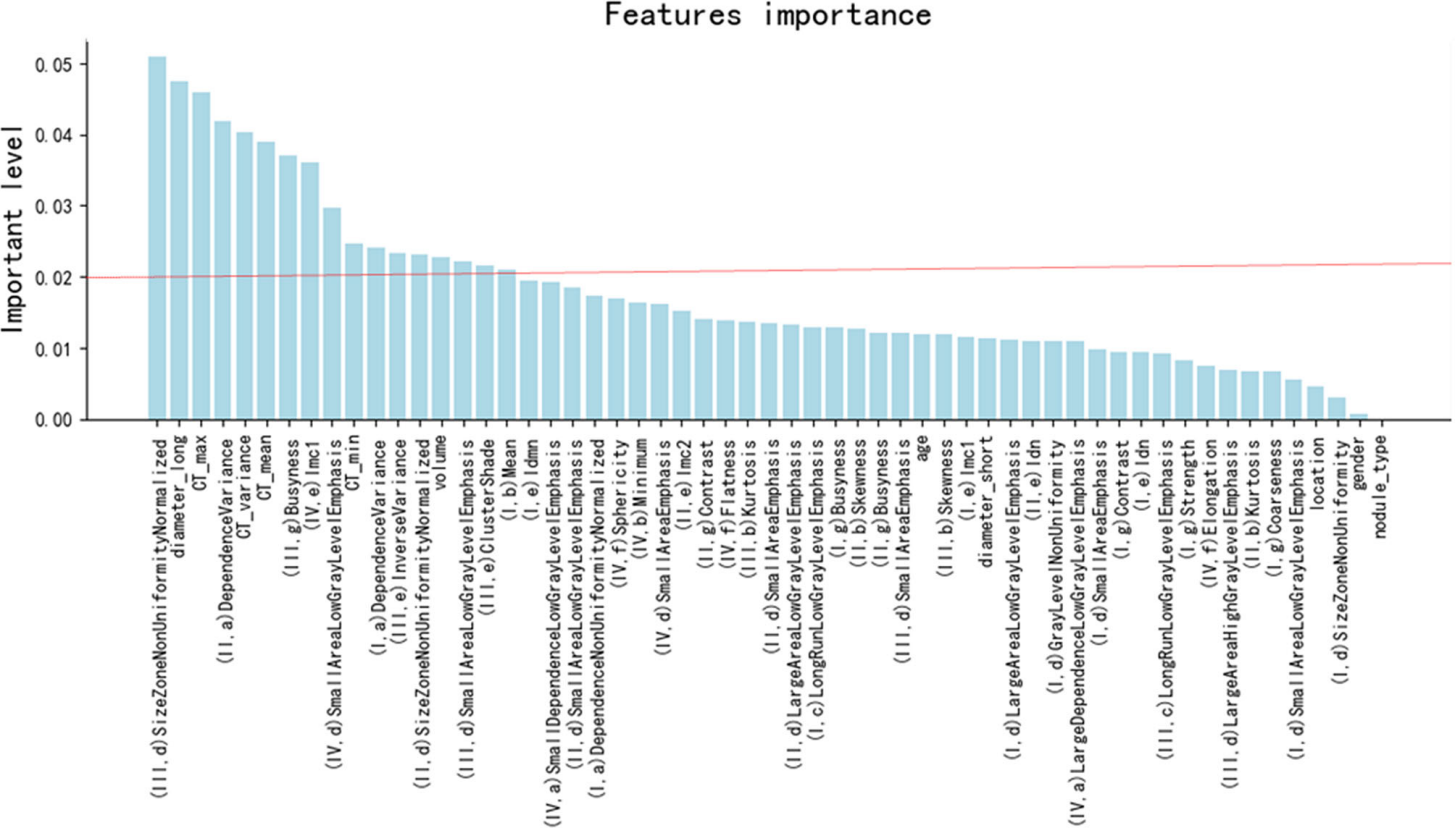
The order of the importance level of the radiomics features and clinical features. I: the filter of log_sigma_5_0_mm, II: the filter of log_sigma_4_0_mm, III: the filter of log_sigma_3_0_mm, IV: from original pictures. a: Gray Level Dependence Matrix features, b: First-Order features, c: Gray Level Run Length Matrix features, d: Gray Level Size Zone Matrix features, e: Gray Level Co-occurrence Matrix feature, f: Shape features, g: Neighboring Gray Tone Difference Matrix features. CT_max, CT_variance, CT_mean, and CT_min indicate maximum CT value, variance of CT value, mean CT value, and minimum CT value, respectively.

Initially, the number of extracted radiomics features was 386. After Kruskal–Wallis tests, correlation tests, and ICC assessment, 46 radiomics features were retained. Then, the random forest method was used for further selection of 57 features, including 46 radiomics features and 11 traditional features (listed in **Table 1**). The 57 features were classified by order of importance using the random forest method (**Figure 2**). Seventeen features (including 11 radiomics and 6 traditional features), the importance level of which surpassed 0.02, were selected to establish a prediction model. The 11 selected radiomics features are shown in **Supplementary Table S1**, and 6 traditional features (long diameter, maximum CT value, variance of CT value, mean CT value, minimum CT value, and volume) are shown in **Table 1**.

### Establishment of a Radiomics Prediction Model

**Figure 3:**
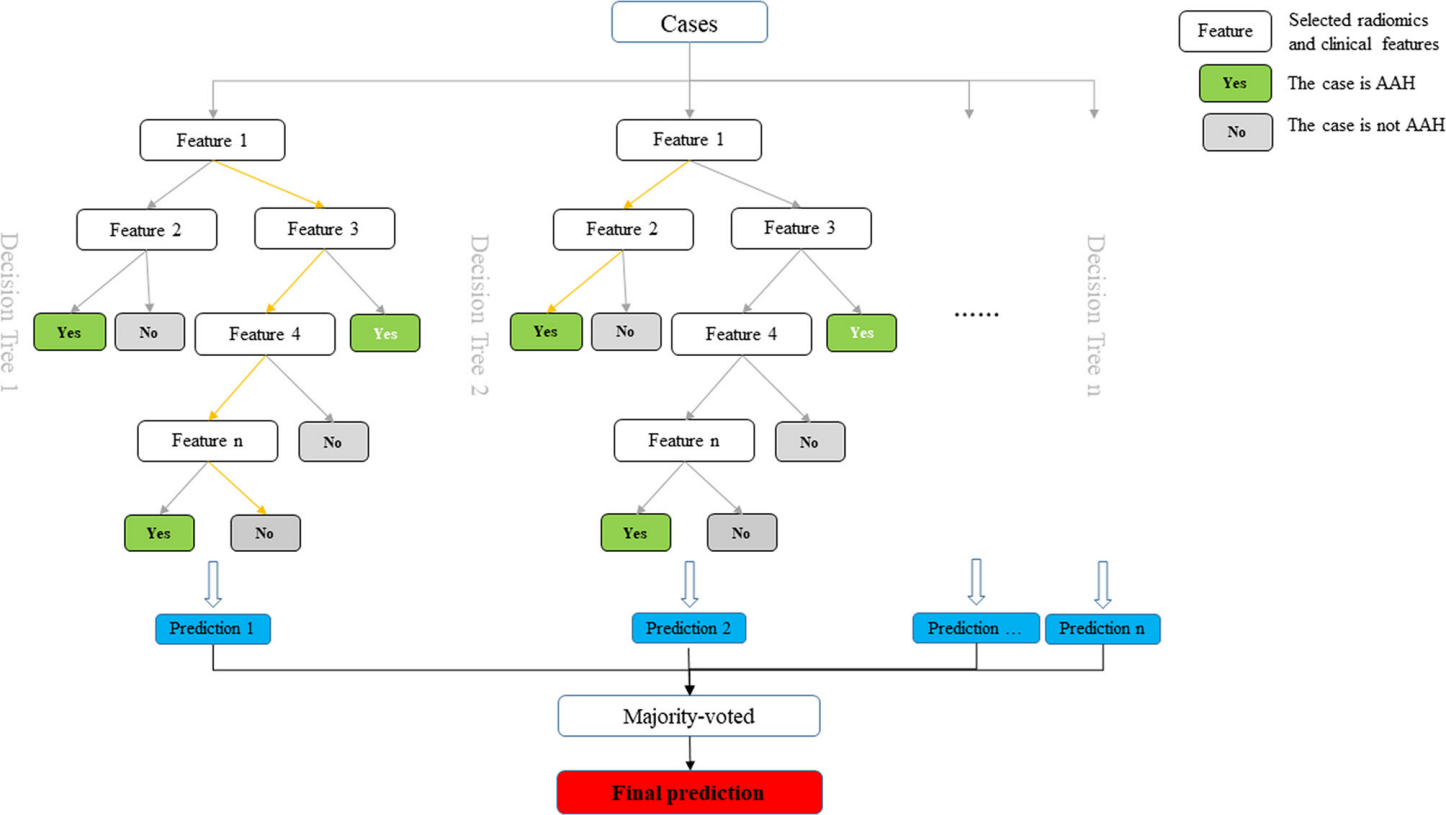
The diagram of established prediction model based on random forest classifier.

We aimed to construct a prediction model to distinguish AAH and adenocarcinomas manifesting as subcentimeter GGNs. The selected 11 radiomics and 6 clinical features were used to construct a prediction model based on the training set, according to the random forest classifier. The performance of the established model was further tested on the validation set. The diagram of the prediction model is shown in **Figure 3**.

### Performance of the Radiomics Prediction Model

**Figure 4:**
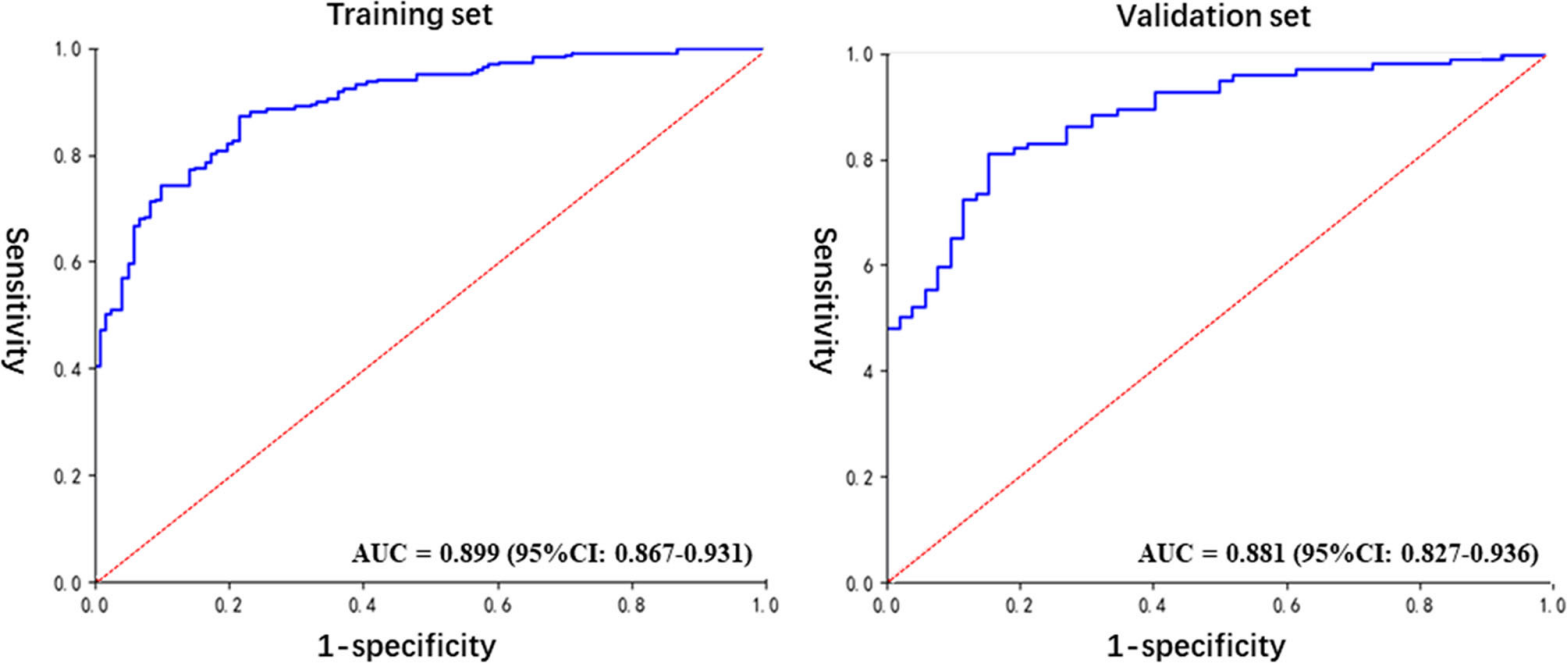
The ROC curves for the diagnosis of the radiomics model in the training and validation sets.

The detailed prediction results obtained with the radiomics model are shown in **Table 2**. The predictive accuracy was 84.1% and 82.2% in the training and the validation set, respectively. The specificity, positive predictive value, and negative predictive values were all over 70% (**Table 3**). The AUC of the prediction model was 0.899 (95% CI: 0.867–0.931) and 0.881 (95% CI: 0.827–0.936) for, respectively, the training and the validation set (**Figure 4**). Compared with the training set, the predictive performance of the radiomics model on the validation set was not significantly lower. These indicators demonstrated that our newly established prediction model performs well to discriminate between AAH and early lung adenocarcinomas with subcentimeter GGNs.

**Table 2:** Confusion matrix of the prediction results obtained from the validation set by the prediction model.

Predictions	Final pathological classification		
	AAH (*n* = 52)	Adenocarcinomas (*n* = 94)	Total (*n* = 146)
AAH	44 (84.6%)	18 (19.1%)	62
Adenocarcinomas	8 (15.4%)	76 (80.9%)	84

Adenocarcinomas include adenocarcinoma in situ, minimally invasive adenocarcinoma, and invasive adenocarcinoma.

AAH, atypical adenomatous hyperplasia.

**Table 3:** Performance of the radiomics model on the training and the validation sets.

Group	Accuracy	Sensitivity	Specificity	PPV	NPV
Training set	84.1%	78.5%	87.2%	77.2%	88.0%
Validation set	82.2%	84.6%	80.9%	71.0%	90.5%

PPV, positive predictive value; NPV, negative predictive value.

## Discussion

Lung cancer is the most common cancer and the primary cause of cancer-related death (19) and adenocarcinomas have been the main histological subtype of lung cancer (20). The widening utilization of chest CT in clinics has reduced the mortality of lung cancer (21, 22), while increasing the detection of many subcentimeter GGNs. Proper management strategy for these detected GGNs is very important for the overall control of lung cancer because of the high prognosis variability linked to different stages of lung adenocarcinomas (23, 24). AAH is considered a precancerous adenocarcinoma lesion. Therefore, accurate discrimination between AAH and adenocarcinomas would be beneficial to the personalized treatment of these two different GGNs. At present, preoperative CT images are the most useful tools to identify the pathologic stage of GGNs. However, a large part of AAH and subcentimeter adenocarcinomas may have only little morphological differences on CT images and are difficult to distinguish for the radiologists. This difficulty may lead to many AAH nodules being treated by aggressive surgical treatment as if they were adenocarcinomas.

Some studies investigated the performance of the radiomics method to predict the pathologic classification of GGNs (16, 17). However, these studies did not try to differentiate AAH nodules from adenocarcinomas. In addition, the diameter of the nodules included in these studies was less than 3 cm, but few AAH nodules had a diameter greater than 1 cm. Consequently, the pathological classification of subcentimeter nodules remained a problem to be solved in clinics. Therefore, we sought to investigate whether a prediction model based on radiomics and clinical features could be used as an effective method to discriminate between these two pathologic types of GGNs.

The accuracy of our newly developed radiomics prediction model was 84.1% and 82.2% in the training and the validation set, respectively (**Table 3**). This accuracy was satisfactory for both sets, and the performance for the validation set displayed no significant reduction compared to that obtained for the training set. These results demonstrated the stability of the prediction model, although further validation is needed. In addition, the sensitivity, specificity, PPV, and NPV were all above 70%, which indicated a good comprehensive performance for the prediction model. Similarly, the analysis by ROC curves revealed AUCs of the prediction model of 0.899 (95% CI: 0.867–0.931) and 0.881 (95% CI: 0.827–0.936) for the training and validation set, respectively (**Figure 4**), indicative of good performance. These indicators proved that our prediction model, based on radiomics and traditional features, is an effective method to distinguish AAH and adenocarcinomas manifesting as subcentimeter GGNs on CT images.

Ranking of the features’ importance level (**Figure 2**) revealed that the radiomics features were as important as the main clinical features. One radiomics feature was in the top three most important features, and four radiomics features were found in the top 10 most important features. These results demonstrate the necessity and meaningfulness of introducing the radiomics method to clinical practice to improve tumor diagnosis.

In this study, one of the most important inclusion criteria was that the diameter of the nodules was less than 1 cm, rather than 3 cm in other studies, to better fit the clinical needs. Preoperative pathological prediction on subcentimeter GGNs is a big challenge for clinicians, because the pathological classification of these lesions, based on different morphological features, is usually difficult to achieved with the naked eye. In this study, our prediction model reached a satisfactory prediction performance, although the included nodules had stricter requirement on the diameter criteria than other studies.

The main limitation of our research was the lack of multicenter data and prospective validation. Therefore, our newly established radiomics classifier will need further stability and applicability validation. Our future research will expand the size of the data set and conduct multicenter clinical trials to verify and improve the effectiveness of the model.

## Conclusion

This study showed that our newly established prediction model performed well in discriminating between AAH and early adenocarcinomas manifesting as subcentimeter lung GGNs on CT images. This model has the potential to improve preoperative prediction accuracy for AAH nodules and may help avoid aggressive surgical treatment for AAH patients. In the future, larger multicenter data and prospective validations will be needed to improve and test the clinical values of this new radiomics classifier.

## Data Availability Statement

The raw data supporting the conclusions of this article will be made available by the authors, without undue reservation.

## Ethics Statement

The studies involving human participants were reviewed and approved by Shanghai Pulmonary Hospital, Tongji University School of Medicine. Written informed consent for participation was not required for this study in accordance with the national legislation and the institutional requirements. Written informed consent was not obtained from the individual(s) for the publication of any potentially identifiable images or data included in this article.

## Author Contributions

The XS and YS proposed the study. BW and PH conducted the research and wrote the manuscript. XM, YY auxiliarily analyzed the data. KS collected and classified the data. All authors contributed to the article and approved the submitted version.

## Funding

This work was supported by the Natural Science Foundation of Shanghai [Grant Number 19ZR1443100] and clinical research project of Shanghai pulmonary hospital [Grant Number fk18007].

## Conflict of Interest

The authors declare that the research was conducted in the absence of any commercial or financial relationships that could be construed as a potential conflict of interest.

## Publisher’s Note

All claims expressed in this article are solely those of the authors and do not necessarily represent those of their affiliated organizations, or those of the publisher, the editors and the reviewers. Any product that may be evaluated in this article, or claim that may be made by its manufacturer, is not guaranteed or endorsed by the publisher.
