# Supplementary material for: Evaluation of the Radiomics Method for the Prediction of Atypical Adenomatous Hyperplasia in Patients With Subcentimeter Pulmonary Ground-Glass Nodules
Source: Front Oncol. 2021 Aug 5;11:698053. doi: 10.3389/fonc.2021.698053 (PMC8374940; doi:10.3389/fonc.2021.698053)
Supplement: Supplementary file 1 [file DataSheet_1.docx]

**Table S1.** Selected radiomics features.

| **Type** | **Name** | | **Calculation formula** | |  |
| --- | --- | --- | --- | --- | --- |
|  |  | |  | |  |
| First Order Features | | | | | |
| Mean* | | 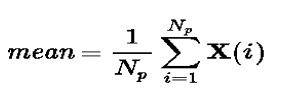 | |  |  |
|  |  | |  | |  |
| GLCM |  | |  | |  |
|  | Inverse Variance* | | 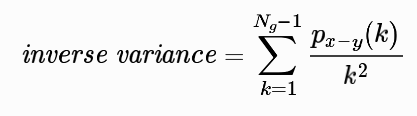 | |  |
|  | Cluster Shade* | | 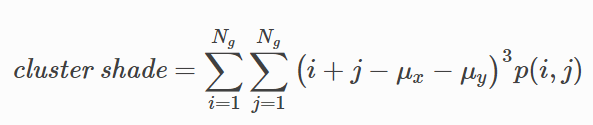 | |  |
|  | IMC 1^#^ | | 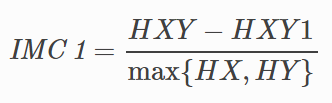 | |  |
| GLSZM |  | |  | |  |
|  | SZNN*^#^ | | 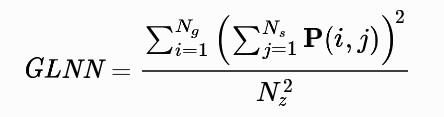 | |  |
|  | SALGLE*^#^ | | 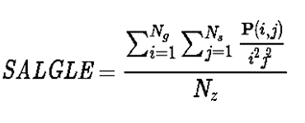 | |  |
| NGTDM |  |  |  |  |  |
|  | Busyness* | | 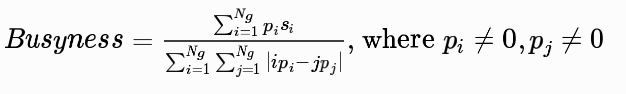 | |  |
| GLDM | DV*^#^ | | 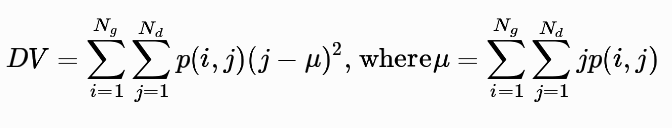 | |  |

NOTE: *, the features were from the filter of logarithm; ^#^, the features were from the filter of wavelet.

(1) First order features.

1)Mean, The average gray level intensity within the ROI.

(2) GLCM, Gray Level Co-occurrence Matrix Features.

1) Inverse Variance, is a measure of the local homogeneity of an image.

2) Cluster Shade, is a measure of the skewness and uniformity of the GLCM.

3) IMC 1, assesses the correlation between the probability distributions.

(3) GLSZM, Gray Level Size Zone Matrix Features Gray Level Co-occurrence Matrix Features.

1) SALGLE, Small Area Low Gray Level Emphasis, measures the joint distribution of small dependence with lower gray-level values.

2) SZNN, Size-zone non-uniformity normalized, it describes the variability of size zone volumes throughout the image.

(4) NGTDM, Neighboring Gray Tone Difference Matrix Features

1) Busyness, it measures the change from a pixel to its neighbor.

(5) GLDM, Gray Level Dependence Matrix Features.

1) Dependence Variance, Measures the variance in dependence size in the image.
