# Supplementary figures and images for: Evaluation of the Radiomics Method for the Prediction of Atypical Adenomatous Hyperplasia in Patients With Subcentimeter Pulmonary Ground-Glass Nodules
Source: Front Oncol. 2021 Aug 5;11:698053. doi: 10.3389/fonc.2021.698053 (PMC8374940; doi:10.3389/fonc.2021.698053)

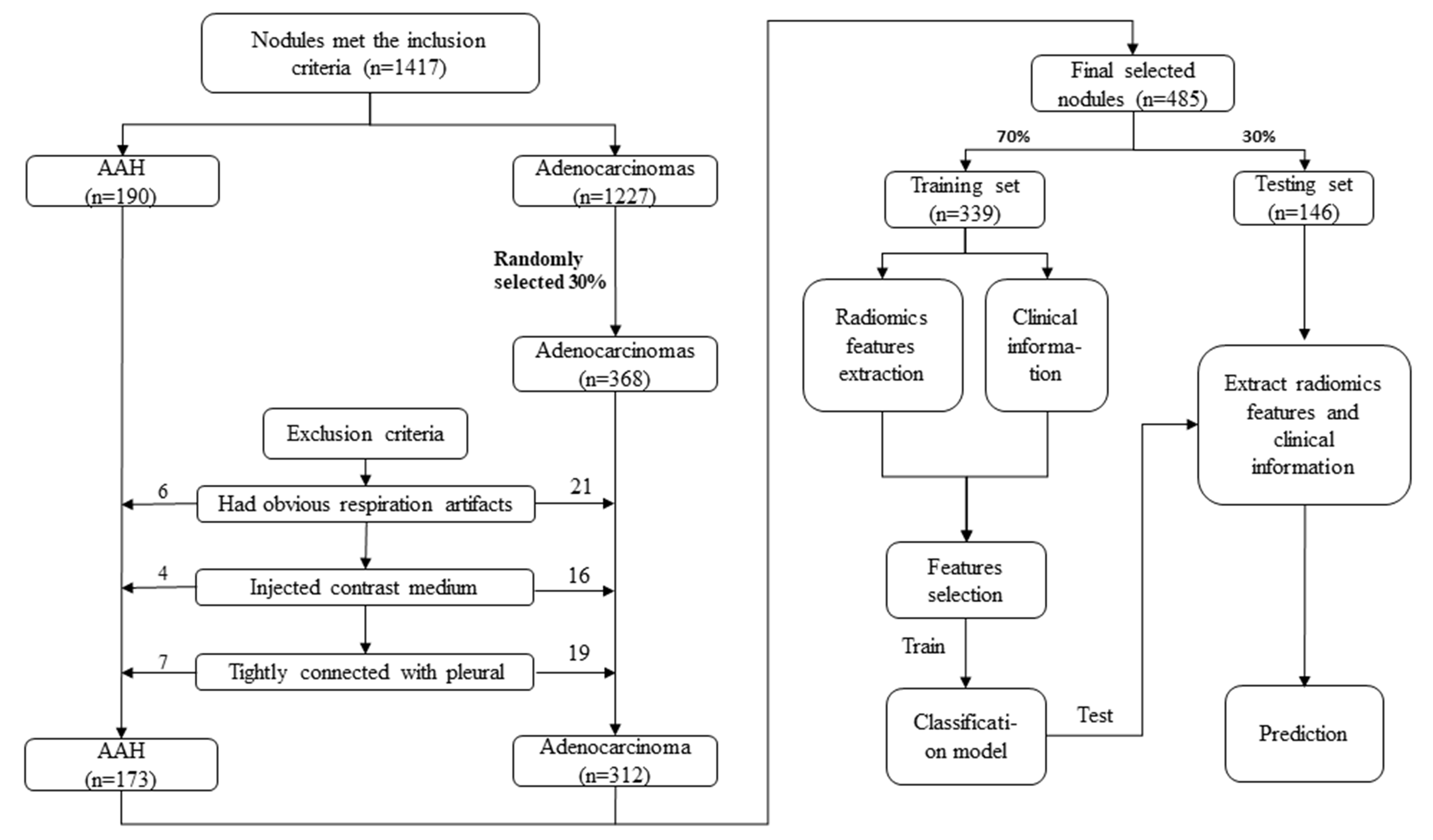

Supplement: Supplementary file 2 [file Image_1.tif]
